# People with HIV on antiretroviral therapy demonstrate robust humoral response to influenza vaccination

**DOI:** 10.1186/s12981-026-00917-x

**Published:** 2026-06-29

**Authors:** Preeti Moar, Pauline A. Ocaya, Christoffer Granvik, Julia Wigren Byström, Koushikul Islam, Niklas Arnberg, Therese Thunberg, Mattias N. E. Forsell

**Affiliations:** 1 Department of Clinical Microbiology, Umeå University, Umeå, 90187 Sweden; 2 Department of Infectious Diseases, Sundsvall Hospital, Sundsvall, Sweden; 3 Science for Life Laboratory, Department of Clinical Microbiology, Umeå University, Umeå, Sweden

**Keywords:** Suppressive ART, Immune restoration, Humoral immunity, Vaccine immunogenicity, Antibody response, Virus neutralization

## Abstract

We evaluated the magnitude and function of antibody responses to seasonal influenza vaccination in individuals with HIV receiving ART compared with individuals without HIV. In a prospective cohort of 78 adults, influenza strain-specific IgG levels were measured up to one-year post-vaccination. Linear mixed-effects models assessed longitudinal trends. IgG levels peaked at two weeks and declined gradually in both groups. HIV status did not significantly influence antibody magnitude or neutralizing activity, and H1N1 antibody levels correlated with neutralization titers. These findings demonstrate robust, durable, and functional humoral response in well-controlled HIV, supporting the effectiveness of annual influenza vaccination in this population.

**Supplementary Information:**

The online version contains supplementary material available at 10.1186/s12981-026-00917-x.

## Introduction

People with human immunodeficiency virus (HIV) infection remain at increased risk for severe outcomes from seasonal influenza despite the widespread availability of effective antiretroviral therapy (ART). Consequently, annual influenza vaccination is recommended for people with HIV (PWH) as part of routine preventive care. While ART restores CD4 T-cell counts and reduces immune activation, residual immune dysfunction may persist [1, 2], potentially influencing vaccine responsiveness [3, 4], therefore, the magnitude and quality of vaccine-induced immune responses in PWH remain subjects of ongoing investigation.

Earlier studies frequently reported diminished or short-lived vaccine responses among PWH, often associated with low CD4 T-cell counts or uncontrolled viremia [5, 6]. Influenza vaccination was historically associated with transient increases in HIV replication [7, 8], and impaired vaccine responses were linked to generalized immune activation, B-cell dysregulation, and altered T follicular helper cell function [9, 10]. However, contemporary evidence shows that effective ART normalizes many aspects of adaptive immunity, enabling serological responses comparable to healthy individuals [11, 12], yet data remain heterogeneous, partly due to differences in study design, participant characteristics, and vaccine formulations.

Evaluating antibody responses to vaccination can provide important insights into the functional recovery of B-cell immunity and understanding the extent of immune restoration in PWH on suppressive ART has direct implications on determining the adequacy of current immunization strategies for this key population. Here we compare the magnitude and durability of humoral immune responses to influenza vaccination in an age and sex-matched longitudinal cohort of PWH on ART and people without HIV (PWOH).

## Methods

### Study design and participants

We conducted a prospective observational cohort study between November 2018 to December 2019 at the infectious disease clinic of Umeå University Hospital. A total of 78 adults were enrolled (*n* = 38 PWH and *n* = 40 PWOH). PWH had been on suppressive ART for at least 6 months before the start of the study. PWOH were included based on the absence of treated chronic diseases and no history of immunotherapy or immunosuppressive treatment. Participants in the two groups were matched based on age and sex, and all participants received a single dose of the 2018–2019 inactivated quadrivalent influenza vaccine formulated for the Northern hemisphere (Supplement S1). Demographic, hematologic and biochemical parameters were obtained from the clinical records. The study was approved by the Swedish Ethical Review Board (DNR 2018-161-31 M; approved 2018-06-17). Informed consent was obtained through oral and written procedures.

### Sample collection and analysis

**Fig. 1:**
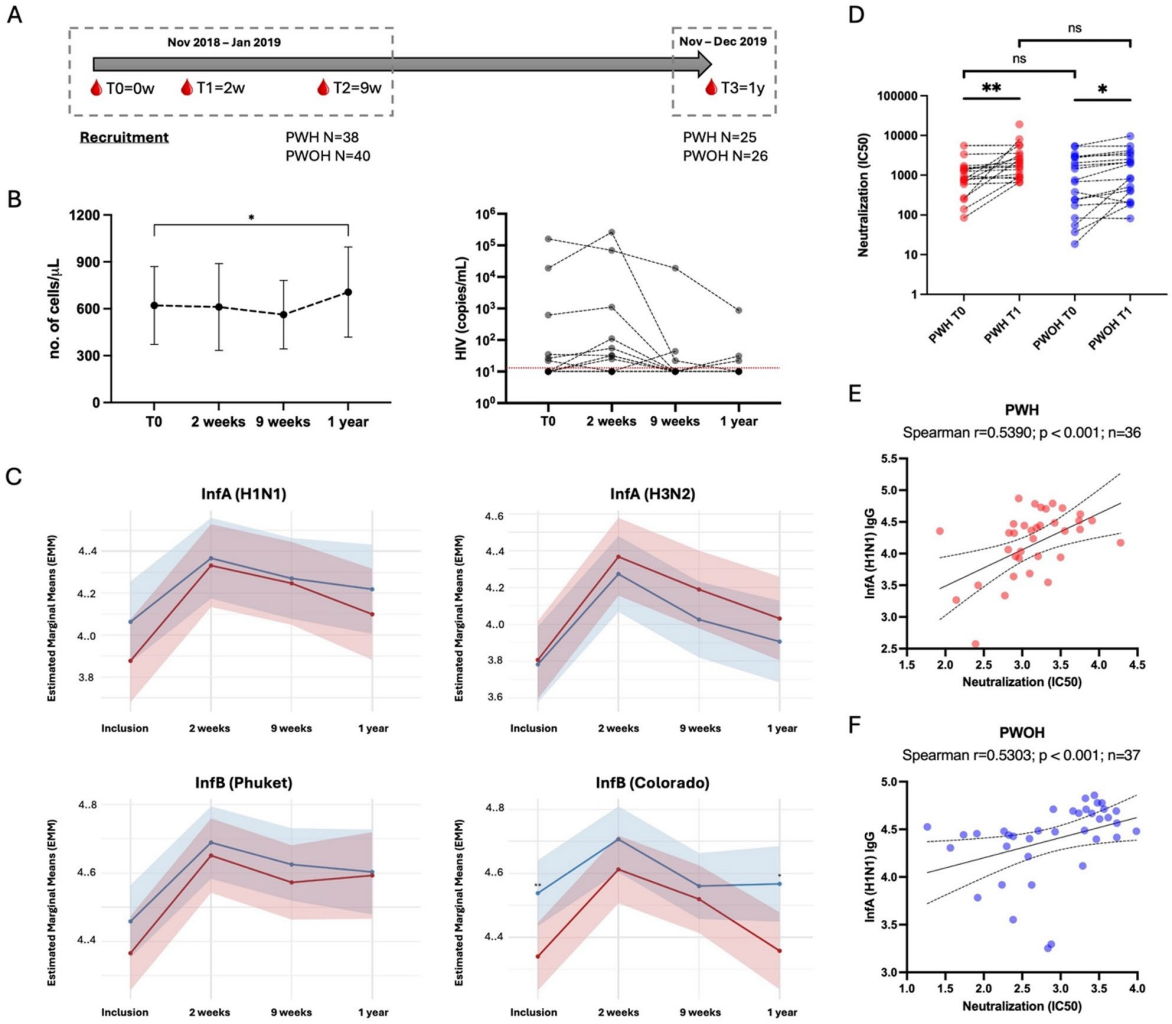
Study timeline and antibody responses following vaccination. **A** Study design and sampling schedule with number of participants included. **B** CD4 T-cell counts and HIV viral load in PWH over the follow-up period. Horizontal red line represents threshold value for detectable HIV RNA. Asterisk (*) represents statistical significance (Wilcoxon rank-sum test; *p* < 0.05). **C** Estimated marginal means of strain-specific IgG levels across follow-up visits post vaccination, assessed using LMMs. Line colors represent HIV status (red = PWH, blue = PWOH), shaded areas represent 95% confidence intervals. Asterisk (*) represents significant differences between PWH and PWOH (*p* < 0.05). **D** Influenza A (H1N1) pdm09 virus neutralization for PWH and PWOH at baseline (T0) and 2 weeks post-vaccination (T1). Dotted lines connect paired samples. Statistical comparisons were performed using paired t-test within groups and Mann Whitney test between groups. Asterisk (*) represents statistical significance (**p < 0.005, *p < 0.05, ns = not significant). **E**–**F** Correlation between neutralization capacity and IgG levels. Log-transformed IC₅₀ values were correlated with log-transformed InfA H1N1-specific IgG levels in PWH and PWOH using Spearman correlation. Lines represent linear regression with 95% confidence intervals

Peripheral blood was collected immediately before vaccination (T0) and at 2 weeks (T1), 9 weeks (T2) and 1 year (T3) post vaccination (Fig. 1A). Plasma was separated and stored at -80 °C for further analysis. CD4 T-cell counts and plasma HIV viral load were measured in PWH. Complete blood counts and clinical chemistry records of all participants were available. Influenza strain-specific IgG levels were quantified using a bead-based multiplex serological assay based on Luminex technology (detailed method in Supplement S2). Neutralization potency was assessed by quantifying the ability of plasma samples to inhibit infection of A549 cells by a clinical isolate of influenza A (H1N1) pdm09 strain (detailed method in Supplement S3). Neutralizing antibody titers were calculated as the half-maximal inhibitory concentration (IC₅₀), defined as the reciprocal plasma dilution required to inhibit 50% of viral infection. To evaluate the relationship between binding and functional responses, associations between IC₅₀ values and strain-specific IgG levels were assessed using Spearman correlation coefficient.

### Statistical analysis and data visualization

Data was analyzed and visualized using GraphPad Prism version 10.5.0 and R versions 4.2.2 and 4.4.1. Continuous variables were summarized as medians with interquartile ranges (IQR) and compared using the Wilcoxon rank-sum test when comparing paired values, and Mann-Whitney test when comparing unpaired values. Categorical variables were summarized as counts and percentages and compared using Fisher’s exact test. Trends in antibody titers across longitudinal sampling time-points were analyzed using linear mixed-effects models (LMMs). We fitted LMMs separately for each of the four influenza strains InfA/Michigan, InfA/Singapore, InfB/Phuket, and InfB/Colorado. The outcome variable in each model was log-transformed influenza strain-specific IgG levels, as measured by the serology assay. To account for the within-subject correlation of repeated measurements, a random intercept for each participant was included in all models. The primary fixed effects included time, HIV status, age and sex. Time was modeled as a categorical variable with four levels: inclusion (T0), 2 weeks (T1), 9 weeks (T2) and 1 year (T3). To explore whether the vaccine response kinetics differed by HIV status, HIV × time interaction terms were included in all models. HIV viral load and CD4 T-cell count were incorporated only as baseline inclusion covariates and not as time-varying variables. LMMs were fitted using the *lme4* package. Estimated marginal means and pairwise comparisons were computed using the *emmeans* package.

## Results

### Participant characteristics and clinical parameters

Participant demographics are summarized in Table 1. A total of 78 participants (PWH *n* = 38; PWOH *n* = 40) with median age 47 years (IQR: 37–56 years) were included in the study. Median age was similar between groups (PWH = 49 years, PWOH = 47 years; *p* = 0.82), and 50% of the total participants were female with similar distribution between groups (Table 1). The median ART duration was 12 years (IQR: 5–16 years) and the majority were treated with NRTI + INSTI based regimens (Table 1). Among, PWH, median baseline CD4 T-cell count was 600 cells/µL (IQR: 411–754 cells/µL) at baseline and improved over time, with significant increase at 1 year follow-up after vaccination (Fig. 1B; *p* < 0.05). Majority (84%) of the participants had < 20 HIV RNA copies/mL at baseline and viral load remained suppressed throughout follow-up except for transient blips 2 weeks post vaccination that were not statistically significant (Fig. 1B).

Routine hematologic and biochemical parameters were largely within normal ranges. Compared with PWOH, PWH had lower baseline leukocytes (5.85 vs. 6.40 × 10^6^/mL, *p* = 0.022), thrombocytes (213 vs. 253 × 10^6^/mL, *p* = 0.004) and neutrophils (2.95 vs. 3.90 × 10^6^/mL, *p* = 0.004), and increased reticulocytes (60 vs. 51 × 10^6^/mL, *p* = 0.048; Table 1). While total leukocytes normalized in the follow-up after vaccination, neutrophils remained low up to 2 weeks post vaccination in PWH compared to PWOH and thrombocytes remained low up to 9 weeks post vaccination. Reticulocytes remained higher in PWH compared to PWOH throughout baseline to 9 weeks post vaccination (Supplement S4). Additionally, higher baseline levels of liver enzymes were observed in PWH (ALP, *p* = 0.014; ASAT, *p* = 0.015; ALAT, *p* = 0.015; LD, *p* = 0.018; Table 1). While LD normalized in the follow-up after vaccination, ASAT and ALAT remained high in PWH compared to PWOH up to 2 weeks post vaccination and ALP up to 9 weeks post vaccination (Supplement S5). Other biochemical markers, including hemoglobin, ferritin, creatinine, and C-reactive protein, did not differ significantly between groups (Table 1).

### Robust and durable humoral response to influenza vaccination in PWH

PWH exhibited strong induction of influenza-specific IgG responses, peaking at 2 weeks post-vaccination (Fig. 1C and Supplement S6). In LMMs, time was the strongest predictor of IgG levels across all four influenza strains (Supplement S6). Sex was not a significant factor influencing antibody responses post vaccination, whereas age had a small negative effect only in the InfA/Singapore model (β= –0.010, *p* = 0.0319). HIV had minimal influence on absolute antibody magnitude after adjusting for age, sex, and repeated measures. At weeks 2 and 9 after vaccination, IgG responses were comparable between PWH and PWOH for all strains (Fig. 1C). HIV status was only a significant predictor for the InfB/Colorado strain at baseline and one year post vaccination. Antibody levels increased at 2 weeks post vaccination, with significant positive coefficients for all four influenza strains (A/Michigan H1N1, A/Singapore H3N2, B/Phuket Yamagata, and B/Colorado Victoria; all *p* < 0.001). Titers remained elevated at 9 weeks and, although attenuated, were still above baseline after one year for most strains (Supplement S6). HIV × time interaction was not significant for any of the strains post vaccination except for the InfB/Colorado strain at the 9-week time-point (Supplement S7).

To determine whether comparable IgG responses translated into functional antibody responses, we evaluated influenza virus neutralization. Neutralizing antibody potency increased following vaccination in both PWH and PWOH (*p* = 0.004 and *p* = 0.01, respectively), with no significant differences in IC₅₀ values between groups across time points (Fig. 1D). IC₅₀ values correlated positively with H1N1-specific IgG levels in both groups (Fig. 1E-F; *p* < 0.001) indicating that higher binding antibody responses were associated with greater neutralization capacity.

## Discussion

This study demonstrates that ART-treated PWH mount robust and durable antibody responses to influenza vaccination comparable to PWOH. Using longitudinal serological profiling, we showed that influenza strain-specific IgG levels peaked two weeks post-vaccination, followed by gradual waning in both groups, consistent with known kinetics of post-vaccination immunity in adults. HIV status did not influence the antibody responses except for a modest difference at one year post vaccination specific to the InfB/Colorado strain. However, we believe this was largely an effect of the difference in baseline antibody levels, presumably due to pre-vaccination exposure to influenza in the PWOH group. Importantly, the observed correlation between IgG binding levels and neutralization titers supports the relevance of antibody levels as a proxy for functional humoral immunity. Our results indicate that influenza vaccination elicits quantitatively and functionally comparable antibody responses in treated PWH relative to PWOH. The durability of antibody responses over one year highlights the presence of functional plasma cells and long-lived memory B-cell responses in well-controlled HIV infection. These findings indicate that effective viral suppression under ART largely restores humoral responsiveness to vaccination, supporting the public health value of annual influenza vaccination in this population. Previous work has shown that aging influences influenza vaccine responses in PWH [13]. While age was adjusted for in our models, the relatively younger cohort may underestimate such effects. Future studies enriched for older adults (> 60 years) will be important to determine whether this observation of comparable antibody responses can be extended to elderly PWH.

In our study, PWH exhibited mild baseline alterations in hematologic and biochemical parameters, that did not correlate with antibody magnitude, suggesting that residual systemic perturbations in treated HIV infection do not meaningfully affect vaccine responsiveness. The persistent elevation in reticulocytes and episodic cytopenias seen in PWH [14, 15] as well as alteration in liver enzymes [16, 17] have been previously reported as consequences of chronic HIV-related inflammation or ART toxicity. Moreover, the degree of viral suppression and immune reconstitution is an important determinant of vaccine responsiveness in PWH. Previous studies have reported impaired humoral responses to influenza vaccination in individuals with ongoing viremia or low CD4 T-cell counts [18]. In contrast, our cohort consisted of individuals on suppressive ART with relatively preserved immune status, and we did not observe a significant association between CD4 T-cell counts or viral load and antibody levels (Supplement S8). Among PWH in this cohort, CD4 T-cell counts remained relatively consistent over time and even improved one year post vaccination. HIV RNA remained undetectable with only transient low-level “blips” post vaccination. Viral blips as an effect of influenza vaccination have been reported before [19]. Despite these temporary blips, 79% of the participants at week 2, 92% at week 9 and 88% at one year post vaccination remained virally suppressed. Our results align with recent reports that sustained viral suppression and CD4 recovery restore effective humoral immune responses in PWH [12, 20]. This may not be generalizable to individuals with incomplete immune recovery or uncontrolled viremia.

Limitations include the modest sample size, while sufficient to detect group-level differences in antibody titers, limited the power for subgroup analyses, such as the effects of specific ART regimens or nadir CD4 counts. Moreover, study design did not include collection of follow-up samples to test pre- or post-vaccination influenza infections which may influence antibody levels. Participants were recruited from a single geographic region with high ART coverage, which may limit generalizability to resource-limited areas or to individuals with incomplete viral suppression. Future studies should investigate the cellular and functional immune correlates of protection, exploring vaccine responsiveness across diverse ART regimens and comorbidities. Glycoprofiling approaches could elucidate qualitative features of vaccine-induced antibodies that influence protective efficacy in chronic HIV infection.

**Table 1:** Participant demographics and baseline clinical parameters

	Overall *N* = 78^1^	PWOH *N* = 40^1^	PWH *N* = 38^1^	*p*-value^2^
Age	47 (37, 56)	47 (36, 60)	49 (37, 56)	0.819
Sex				0.497
F	39 (50%)	22 (55%)	17 (45%)	–
M	39 (50%)	18 (45%)	21 (55%)	–
Baseline VL (copies/mL)	–	NA	< 20 (84%)	–
HIV duration (years)	–	NA	13 (8, 18)	–
ART duration (years)	–	NA	12 (5, 16)	–
ART regimen				
NNRTI+NRTI	–	NA	4 (10.5%)	–
NRTI+INSTI	–	NA	23 (60.5%)	–
NRTI + PI	–	NA	9 (23.7%)	–
NRTI+INSTI + PI	–	NA	2 (5.3%)	–
CD4 T-cells (cells/µL)	–	–	600 (411, 754)	–
Potassium (mmol/L)	3.90 (3.80, 4.20)	3.95 (3.80, 4.20)	3.90 (3.80, 4.20)	0.378
Sodium (mmol/L)	139.00 (138.00, 141.00)	139.00 (138.00, 140.00)	139.00 (137.00, 141.00)	0.442
Hemoglobin (g/L)	138 (127, 147)	140 (127, 146)	134 (127, 148)	0.803
Leukocytes (10^6^/mL)	6.20 (5.20, 7.20)	6.40 (5.80, 7.40)	5.85 (4.90, 6.60)	**0.022**
Thrombocytes (10^6^/mL)	245 (195, 281)	253 (231, 303)	213 (188, 263)	**0.004**
Monocytes (10^6^/mL)	0.50 (0.40, 0.60)	0.50 (0.40, 0.60)	0.40 (0.40, 0.60)	0.187
Lymphocytes (10^6^/mL)	1.90 (1.70, 2.30)	1.90 (1.60, 2.40)	1.90 (1.70, 2.20)	0.923
Neutrophils (10^6^/mL)	3.50 (2.70, 4.30)	3.90 (3.00, 4.40)	2.95 (2.20, 4.00)	**0.004**
Ret-MCH (pg)	34.00 (32.00, 35.00)	33.00 (32.00, 34.00)	34.00 (32.00, 36.00)	0.081
Reticulocytes (10^6^/mL)	57 (45, 68)	51 (44, 64)	60 (48, 75)	**0.048**
Ferritin (µg/L)	79 (35, 148)	79 (31, 136)	76 (41, 148)	0.810
Transferrin saturation (%)	24 (20, 32)	25 (21, 31)	24 (18, 34)	> 0.999
ALP (µkat/L)	1.20 (1.00, 1.40)	1.10 (0.95, 1.25)	1.25 (1.10, 1.60)	**0.014**
ASAT (µkat/L)	0.39 (0.32, 0.46)	0.37 (0.31, 0.44)	0.42 (0.35, 0.49)	**0.015**
ALAT (µkat/L)	0.34 (0.29, 0.46)	0.30 (0.27, 0.44)	0.37 (0.30, 0.50)	**0.015**
Bil-total (µmol/L)	6.0 (5.0, 9.0)	6.0 (5.0, 10.5)	6.5 (4.0, 9.0)	0.774
Bil-conjugated (µmol/L)				0.234
< 2	76 (93.6%)	39 (97.5%)	34 (89.5%)	
LD (µkat/L)	3.60 (3.30, 4.00)	3.50 (3.10, 3.90)	3.70 (3.40, 4.20)	**0.018**
GT (µkat/L)	0.30 (0.20, 0.60)	0.30 (0.20, 0.45)	0.40 (0.30, 0.70)	0.052
CRP (mg/L)	1.10 (0.60, 3.10)	0.90 (0.60, 2.20)	1.20 (0.60, 3.30)	0.322
Creatinine (µmol/L)	71 (63, 85)	71 (64, 79)	77 (62, 90)	0.342

Summary of baseline demographic, hematologic, and biochemical characteristics among PWH on suppressive ART and PWOH. Data are presented as median (Q1–Q3) or n (%). p-values calculated using Wilcoxon rank sum test or Fisher’s exact test as appropriate

*VL* Viral Load, *NNRTI* Non-Nucleoside Reverse Transcriptase Inhibitors, *NRTI* Nucleoside Reverse Transcriptase Inhibitors, *INSTI* Integrase Strand Transfer Inhibitors, *PI* Protease Inhibitors, *MCH* Mean Corpuscular Hemoglobin, *ALP* Alkaline Phosphatase, *ASAT* Aspartate Aminotransferase, *ALAT* Alanine Aminotransferase, *Bil* Bilirubin, *LD* Lactate Dehydrogenase, *GT* Gamma-Glutamyl Transferase, *CRP* C-reactive protein

Units: L=Liters, mg=milligram, mL=milliliter, pg=picogram, µg=microgram, µkat/L≈60U/L, µL=microliter

^1^Median (Q1, Q3); n (%)

^2^Wilcoxon rank sum test, Fisher’s exact test

## Conclusion

We conclude that PWH on suppressive ART generate strong and durable antibody responses to seasonal influenza vaccination. Although minor hematologic differences were observed, antibody response to all vaccine strains remained robust and persisted for one year, indicating preserved long-term antibody function in well-controlled HIV infection. Importantly, binding antibody levels were accompanied by comparable functional activity across groups. Sustained viral suppression and immune reconstitution likely underpin these responses. Moreover, we show that standard-dose inactivated influenza vaccines are immunogenic, regardless of HIV status. Together, these findings underscore the effectiveness of ART-mediated immune recovery and support continued annual influenza vaccination as a key component of preventive care for key populations.

## Supplementary Information

Below is the link to the electronic supplementary material.

Supplementary Material 1

## Data Availability

Data sharing is regulated by the General Data Protection Regulation 2016/679, the Swedish Law on Biobanking and approved ethical permits. The study data comprises identifiable and sensitive information that cannot be shared due to protection of personal integrity of the participants.

## Abbreviations

ART: Antiretroviral therapy
ALAT: Alanine aminotransferase
ALP: Alkaline phosphatase
ASAT: Aspartate aminotransferase
CD4: Cluster of differentiation 4
HA: Hemagglutinin
HIV: Human immunodeficiency virus
IC_50_: Half-maximal inhibitory concentration
INSTI: Integrase strand transfer inhibitors
IQR: Interquartile range
LD: Lactate dehydrogenase
LMM: Linear mixed-effects model
NRTI: Nucleoside reverse transcriptase inhibitors
PWH: People with HIV
PWOH: People without HIV
