# Supplementary material for: People with HIV on antiretroviral therapy demonstrate robust humoral response to influenza vaccination
Source: AIDS Res Ther. 2026 Jun 29;23:66. doi: 10.1186/s12981-026-00917-x (PMC13340008; doi:10.1186/s12981-026-00917-x)
Supplement: Supplementary file 1 — Supplementary Material 1 [file 12981_2026_917_MOESM1_ESM.pdf]

S1. Inactivated quadrivalent influenza vaccine formulation (2018-2019)

The Northern hemisphere vaccine contained the following strains:

Influenza A/Michigan/45/2015 (H1N1) pdm09-like virus
Influenza A/Singapore/INFIMH-16-0019/2016 (H3N2)-like virus
Influenza B/Phuket/3073/2013-like virus (B/Yamagata/16/88 lineage)
Influenza B/Colorado/06/2017-like virus (B/Victoria/2/87 lineage).

S2. Detailed methodology for multiplex serology assay

The following hemagglutinin (HA) antigens were used corresponding to the influenza strains included in the quadrivalent vaccine:

Antigen	Company	Cat#
Influenza A H1N1 (A/Michigan/45/2015) Hemagglutinin / HA Protein (His Tag)	Sino Biological	40567-V08H1
Influenza A H3N2 (A/Singapore/INFIMH-16-0019/2016) Hemagglutinin / HA Protein (His Tag)	Sino Biological	40580-V08H
Influenza B (B/PHUKET/3073/2013) Hemagglutinin / HA Protein (His Tag)	Sino Biological	40498-V08B
Influenza B (B/Colorado/06/2017) Hemagglutinin / HA Protein (His Tag)	Sino Biological	40581-V08H

HA antigens were coupled to magnetic MagPlex 6.5 µm COOH-microspheres (Luminex Corporation) following the manufacturer's protocols (xMAP Cookbook, 6th edition). Antigen-coupled microspheres were the added to a 96-well flat bottom black plate (Cat# 237108, Thermo Scientific) at a concentration of 500 microspheres of each set/well in a total volume of 50 µL Phosphate Buffered Saline with 0.05% Tween-20 (PBS-T) containing 0.1% bovine serum albumin and 0.05% sodium azide (PBS-TBN). Plasma samples diluted 1/200 in a total volume of 50 µL/well were added to the antigen-coupled microspheres mixture and incubated at room temperature for 1 h on a microplate shaker (Thermo Scientific) at 800 rpm protected from light. Longitudinal samples from each participant were added on the same plate for consistency. Duplicate blank wells containing only antigen-coupled microspheres in PBS-TBN were also added to monitor background. After the first incubation, plates were washed thrice with 200 µL/well of PBS-TBN, using an automated plate washer with magnetic adaptor (BioTek 406 with BioStack 3WR, Agilent Technologies). Further, 50 µL of 1 µg/mL anti-human IgG Fc-PE (Cat# 9040-09, Southern Biotech) diluted in PBS-TBN was added to all wells and incubated at RT for 1 h at 800 rpm protected from light. Plates were washed three times and beads were resuspended in 60 µL of PBS-TBN for analyzing on the Intelliflex DR-SE (Luminex Corporation) with 40 µL of acquisition volume per well and doublet discriminator gate set at 7000–20000. The outcome was median fluorescence intensity (MFI) from 50 microspheres. Raw data was exported in the form of a CSV file using the in-built xPONENT software.

S3. Detailed methodology for influenza virus neutralization assay

Human lung adenocarcinoma basal epithelial cells, A549, were maintained in DMEM (Sigma-Aldrich) supplemented with 2g NaHCO3/L, 5% fetal bovine serum (FBS, Cytiva) and penicillin G (100 IU/mL) and streptomycin sulfate (100 µg/mL) combined (1× PEST, Gibco) at 37°C and 5% CO2. Neutralization activity of the participant plasma against InfA-H1N1pdm09 virus was conducted using immunofluorescence-based assay as described previously [1]. Briefly, one day before infection, A549 cells were seeded in 96 well plates (approximately 2.2×10^4 cells/well) supplemented with cell maintaining media. Plasma samples were serially diluted 5-fold and mixed with approximately 1100 FFUs of InfA-H1N1pdm09 in serum free DMEM, with the aim to have a multiplicity of infection (MOI) of 0.05. This mixture was incubated for 30 minutes at 37°C. The cells were then infected with the virus and plasma mixture and incubated for 18h at 37°C with 5% CO2. Cells were fixed with 4% formaldehyde and permeabilized in PBS containing 0.1% trition-X-100. IAV-H1N1pdm09 foci were detected using primary rabbit monoclonal antibodies directed against virus nucleoprotein (1:1000, Cat# MA5-42364, Thermo Scientific) and secondary anti-rabbit 1:1000, (A-31573, Cat# Thermo Scientific); cellular nuclei were stained with 0.1% DAPI for 10 min. Antibody-bound infection foci and cell nucleus were visualized and counted using Cytation 5 imager (Agilent BioTek) and virus infection was determined as % of virus control (without addition of patient plasma).

Reference:

1. Islam K, Carlsson M, Enquist P-A, Qian W, Marttila M, Strand M, et al. Structural Modifications and Biological Evaluations of Rift Valley Fever Virus Inhibitors Identified from Chemical Library Screening. ACS Omega [Internet]. American Chemical Society; 2022 [cited 2026 Jun 10];7:6854–68. https://doi.org/10.1021/acsomega.1c06513

S4. Hematologic parameters over the follow-up period post vaccination. Line colors represent HIV status (red = PWH, blue = PWOH) and asterisks (*) represent significant differences between PWH and PWOH (Mann-Whitney test; *p < 0.05, **p < 0.01)

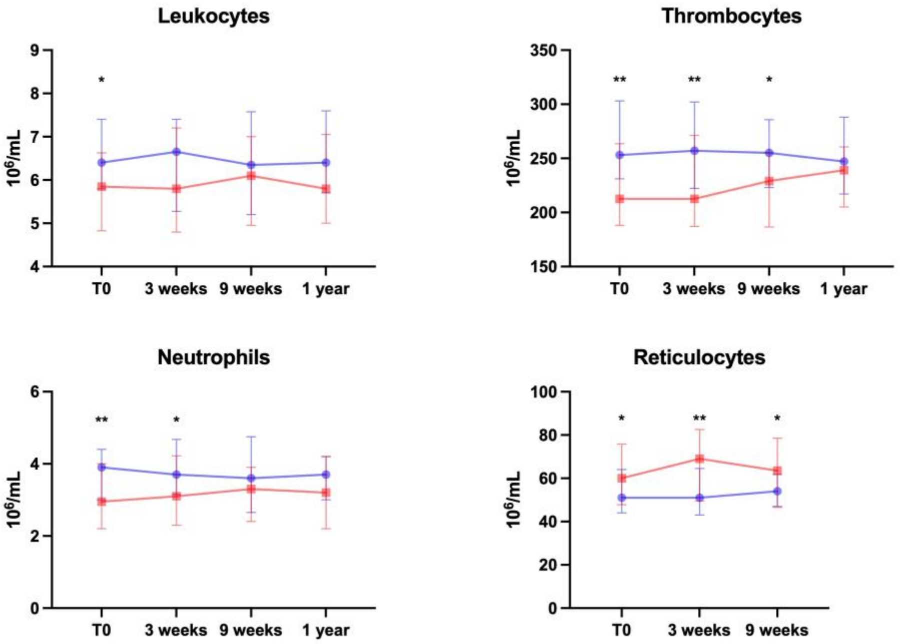

S5. Liver enzymes over the follow-up period post vaccination. Line colors represent HIV status (red = PWH, blue = PWOH) and asterisks (*) represent significant differences between PWH and PWOH (Mann-Whitney test; *p < 0.05, **p < 0.01)

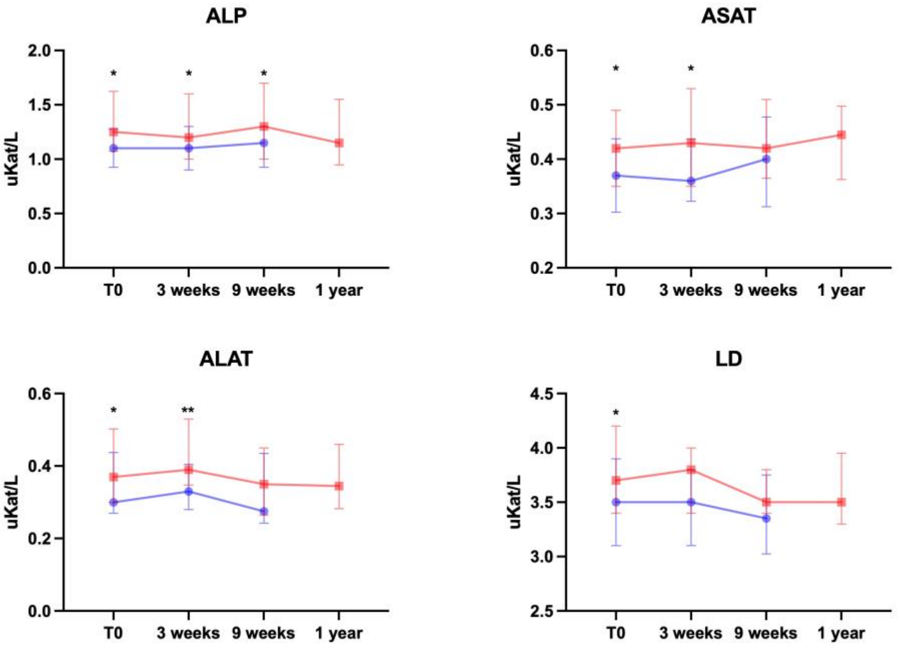

S6. Summary of linear LMMs to evaluate the trends in influenza strain-specific IgG levels at different sampling points post vaccination.

Variable	InfB Phuket IgG	InfB Colorado IgG	InfA Michigan IgG	InfA Singapore IgG
(Intercept)	4.47 (4.26–4.67) p=9.45e-60	4.45 (4.24 – 4.66) p=1.1e-57	3.94 (3.51–4.36) p=9.02e-31	4.21 (3.73–4.68) p=2.4e-29
2 weeks	0.26 (0.18–0.34) p=5.52e-09	0.22 (0.15 – 0.30) p=3.23e-08	0.38 (0.26–0.49) p=7.3e-10	0.53 (0.42–0.63) p=5.44e-19
9 weeks	0.19 (0.11–0.27) p=1.7e-05	0.10 (0.03 – 0.17) p=0.0102	0.29 (0.17–0.40) p=1.87e-06	0.31 (0.21–0.42) p=1.98e-08
1 year	0.19 (0.09–0.28) p=0.00016	0.02 (-0.06 – 0.11) p=0.595	0.19 (0.06–0.32) p=0.00543	0.17 (0.05–0.29) p=0.00505
HIV	-0.05 (-0.16–0.06) p=0.35	-0.13 (-0.24–-0.01) p=0.0288	-0.09 (-0.32–0.15) p=0.456	0.10 (-0.16–0.36) p=0.45
Age	0.00 (-0.00–0.00) p=0.933	0.00 (-0.00 – 0.01) p=0.486	0.00 (-0.01–0.01) p=0.82	-0.01 (-0.02–-0.00) p=0.0319
Male Sex	-0.03 (-0.14–0.08) p=0.557	-0.02 (-0.14–0.10) p=0.725	0.07 (-0.17–0.30) p=0.581	0.04 (-0.23–0.30) p=0.789

S7. Summary of LMMs including HIV × time interactions.

Variable	InfB Phuket IgG Estimate (CI)	InfB Colorado IgG Estimate (CI)	InfA Michigan IgG Estimate (CI)	InfA Singapore IgG Estimate (CI)
(Intercept)	4.48 (4.27-4.69) p= 3.55e-63	4.48 (4.27-4.7) p= 4.16e-60	3.99 (3.56-4.42) p= 1.72e-31	4.24 (3.76-4.71) p= 8.59e-30
Age	0 (0-0) p= 0.934	0 (0-0.01) p= 0.489	0 (-0.01-0.01) p= 0.82	-0.01 (-0.02-0) p= 0.032
HIV (Y)	-0.09 (-0.24-0.06) p= 0.219	-0.2 (-0.34--0.05) p= 0.00747	-0.19 (-0.46-0.08) p= 0.175	0.02 (-0.26-0.31) p= 0.869
Sex (Male)	-0.03 (-0.14-0.08) p= 0.567	-0.02 (-0.14-0.09) p= 0.706	0.06 (-0.17-0.3) p= 0.584	0.04 (-0.23-0.3) p= 0.786
1 year	0.14 (0.01-0.28) p= 0.0325	0.03 (-0.09-0.15) p= 0.628	0.16 (-0.03-0.34) p= 0.0957	0.12 (-0.04-0.29) p= 0.147
1 year:HIV	0.08 (-0.11-0.27) p= 0.39	-0.01 (-0.18-0.16) p= 0.896	0.07 (-0.2-0.33) p= 0.617	0.1 (-0.14-0.34) p= 0.407
2 weeks	0.23 (0.11-0.35) p= 0.000121	0.17 (0.07-0.27) p= 0.00148	0.3 (0.14-0.46) p= 0.000228	0.49 (0.34-0.64) p= 2.85e-10
2w:HIV	0.05 (-0.11-0.22) p= 0.515	0.1 (-0.04-0.25) p= 0.168	0.15 (-0.08-0.38) p= 0.194	0.05 (-0.14-0.28) p= 0.512
9 weeks	0.17 (0.05-0.28) p= 0.00514	0.02 (-0.08-0.13) p= 0.672	0.21 (0.05-0.37) p= 0.0112	0.24 (0.1-0.39) p= 0.00118
9w:HIV	0.04 (-0.13-0.21) p= 0.632	0.16 (0.01-0.31) p= 0.0371	0.16 (-0.07-0.39) p= 0.162	0.14 (-0.07-0.35) p= 0.19

S8. Summary of LMMs using baseline CD4 and viral load as fixed effects.

Model population (PWH only)

Variable	InfB Phuket Estimate (SE)	InfB Colorado Estimate (SE)	InfA Michigan Estimate (SE)	InfA Singapore Estimate (SE)
Intercept	4.202 (0.233) ***	3.963 (0.233) ***	3.244 (0.430) ***	3.223 (0.474) ***
2 weeks	0.480 (0.162) **	0.337 (0.122) **	0.891 (0.226) ***	0.989 (0.199) ***
9 weeks	0.183 (0.103)	0.227 (0.075) **	0.420 (0.139) **	0.401 (0.121) **
1 year	0.204 (0.094) *	-0.006 (0.068)	0.226 (0.126)	0.229 (0.109) *
Baseline CD4	0.00017 (0.00020)	0.000085 (0.00019)	0.00011 (0.00035)	0.00015 (0.00036)
Age	0.00229 (0.00415)	0.00852 (0.00429)	0.01279 (0.00793)	0.00891 (0.00893)
Male Sex	-0.0967 (0.115)	-0.166 (0.116)	-0.0735 (0.214)	0.0302 (0.237)

Variable	InfB Phuket Estimate (SE)	InfB Colorado Estimate (SE)	InfA Michigan Estimate (SE)	InfA Singapore Estimate (SE)
Intercept	4.458 (0.101) ***	4.409 (0.106) ***	3.894 (0.210) ***	4.235 (0.233) ***
2 weeks	0.261 (0.042) ***	0.223 (0.038) ***	0.374 (0.058) ***	0.523 (0.053) ***
9 weeks	0.186 (0.042) ***	0.099 (0.038) *	0.286 (0.058) ***	0.311 (0.053) ***
1 year	0.179 (0.048) *	0.018 (0.044)	0.191 (0.067) **	0.176 (0.061) **
Viral Load ≥20	-0.147 (0.075)	-0.127 (0.071)	0.084 (0.115)	0.053 (0.108)
Age	-0.0003 (0.0019)	0.001 (0.0020)	0.0009 (0.0041)	-0.0097 (0.0045) *
Male Sex	-0.035 (0.056)	-0.028 (0.058)	0.058 (0.118)	0.042 (0.131)
